# Cerebral Toxoplasmosis in a Rheumatoid Arthritis Patient on Immunosuppressive Therapy

**DOI:** 10.7759/cureus.8547

**Published:** 2020-06-10

**Authors:** Brittany Hill, Nicole Wyatt, David Ennis

**Affiliations:** 1 Internal Medicine, Brookwood Baptist Medical Center, Birmingham, USA; 2 Infectious Disease, University of Alabama at Birmingham, Birmingham, USA

**Keywords:** rheumatoid arthritis, toxoplasmosis, immunosuppression, neurology, infectious disease

## Abstract

Cerebral toxoplasmosis is a life-threatening infection most commonly found in immunocompromised hosts such as acquired immunodeficiency syndrome (AIDS) or transplant patients. However, it is not known to affect patients with chronic inflammatory disorders on immunosuppressive therapy. We describe the case of a 70-year-old female with rheumatoid arthritis (RA) on chronic therapy with methotrexate and infliximab, who presented to the hospital after two weeks of right-sided weakness. Imaging revealed bilateral ring-enhancing lesions in the basal ganglia (left greater than right). A diagnosis of cerebral toxoplasmosis was made on brain biopsy. Apart from the immunosuppressive therapy and owning a cat, she had no other risk factors for developing the infection. The patient’s immunosuppressive medications were discontinued, and she was started on high-dose trimethoprim-sulfamethoxazole (TMP-SMX). Upon literature review using PubMed, we found seven other published reports on similar cases of toxoplasmosis in RA patients on immunosuppressive therapy; however, there was a lack of recommendations for diagnosis, treatment, and prophylaxis in this patient population. With the growing use of immunosuppressive therapies in chronic inflammatory disorders, further data is needed regarding the management of toxoplasmosis in these patients. This case report is an investigation of the relationship between immunosuppressive medications in RA patients and cerebral toxoplasmosis and an exploration of the available recommendations for its management.

## Introduction

Toxoplasmosis is one of the most prevalent infections worldwide, affecting an estimated one-third of the world’s population [1]. This infection is caused by *Toxoplasma gondii*, an intracellular protozoan parasite that is usually acquired during childhood and adolescence, and primarily transmitted to humans through ingestion of infectious oocytes, typically from infected cat feces or undercooked meat from an infected animal [2]. It can also be transmitted to a fetus when the mother is infected with the parasite for the first time during pregnancy, resulting in congenital toxoplasmosis [2]. Although the primary infection is asymptomatic or presents as a mild self-limited disease in most immunocompetent hosts, a latent infection can persist for the duration of the host’s life [1]. Reactivation of the parasite, particularly in the immunocompromised, can cause life-threatening disease, most commonly with a brain and eye involvement [2]. Diagnosis of toxoplasmosis encephalitis is dependent on a mix of clinical, serological, and radiological methods. As serologic testing cannot differentiate between a reactivated vs latent infection, most definitive diagnoses are made via polymerase chain reaction (PCR) of the cerebral spinal fluid (CSF) or brain biopsy [1,3]. Treatment of this infection is typically pyrimethamine and sulfadiazine for at least six weeks; however, other medications can also be used, such as trimethoprim-sulfamethoxazole (TMP-SMX) or clindamycin [3].

Although toxoplasmosis is well known in acquired immunodeficiency syndrome (AIDS) patients and other profoundly immunosuppressed hosts such as solid organ or stem cell transplants, there is little data regarding the potential risk for toxoplasmosis in patients undergoing immunosuppressive treatment for inflammatory disorders, specifically with tumor necrosis factor-a (TNF-a) inhibitors [4]. The following case report describes a rheumatoid arthritis (RA) patient with cerebral toxoplasmosis who was on chronic therapy with methotrexate and infliximab. The literature published over the previous 20 years was reviewed using a PubMed search containing the words “toxoplasmosis" and "rheumatoid arthritis”. This search yielded seven published case reports regarding toxoplasmosis in RA patients on immunosuppressive therapy.

## Case presentation

A 70-year-old Caucasian female presented to the emergency department complaining of right-sided weakness. The patient described the weakness as progressive in nature that had begun two weeks prior. One week after the onset of her initial weakness, she had begun to suffer from minor falls due to the right hemiparesis. Her family was present at the bedside and noted that they had observed a mild left-sided facial droop and slurred speech several days before. She denied any head trauma or confusion; however, she admitted to mild right-hand tremors that had started one month prior. Her past medical history was significant for RA, non-insulin-dependent diabetes mellitus, hyperlipidemia, and hypertension. She was on chronic therapy for RA with methotrexate (7.5 mg PO once every week) and infliximab (3 mg/kg IV every eight weeks) for the past two years. Her family and social history were noncontributory, apart from her owning a cat.

On physical examination, she was alert and oriented to person, place, and time. Cranial nerves II-XII were intact, and pupils were 3 mm and reactive. Both upper and lower extremity motor strength was 5/5 on the left and 4/5 on the right. There were no tremors or facial droop noted at the time of the exam.

**Figure 1:**
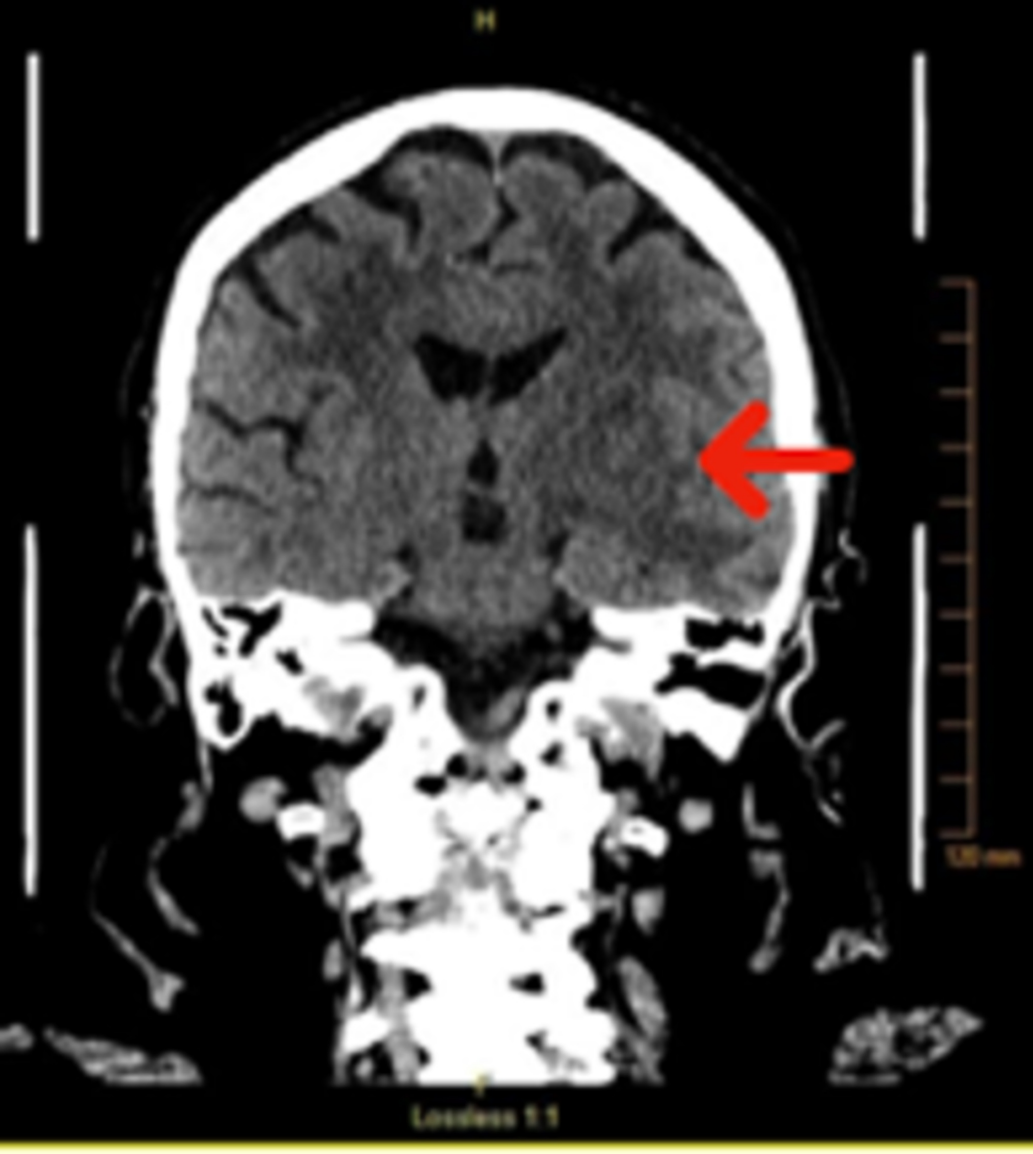
CT of the head without contrast The image showed evidence of bilateral edema and lesions throughout the basal ganglia (left greater than the right, red arrow). There was also mild mass effect on the left lateral ventricle CT: computed tomography

The patient’s complete blood count revealed a white blood cell count of 8,000/uL, platelet count of 266,000/uL, and hemoglobin level of 12.7 g/dL. Her blood chemistry lab work revealed sodium of 143 mEq/L, potassium of 4.2 mEq/L, chloride of 105 mEq/L, carbon dioxide of 27 mEq/L, blood urea nitrogen (BUN) of 24 mg/dL, and creatinine of 0.68 mg/dL. Her glucose level was 91 mg/dL. The patient underwent a head CT without contrast, which demonstrated bilateral edema and lesions throughout the basal ganglia (left greater than right) with mild mass effect on the left ventricle as shown in Figure 1.

**Figure 2:**
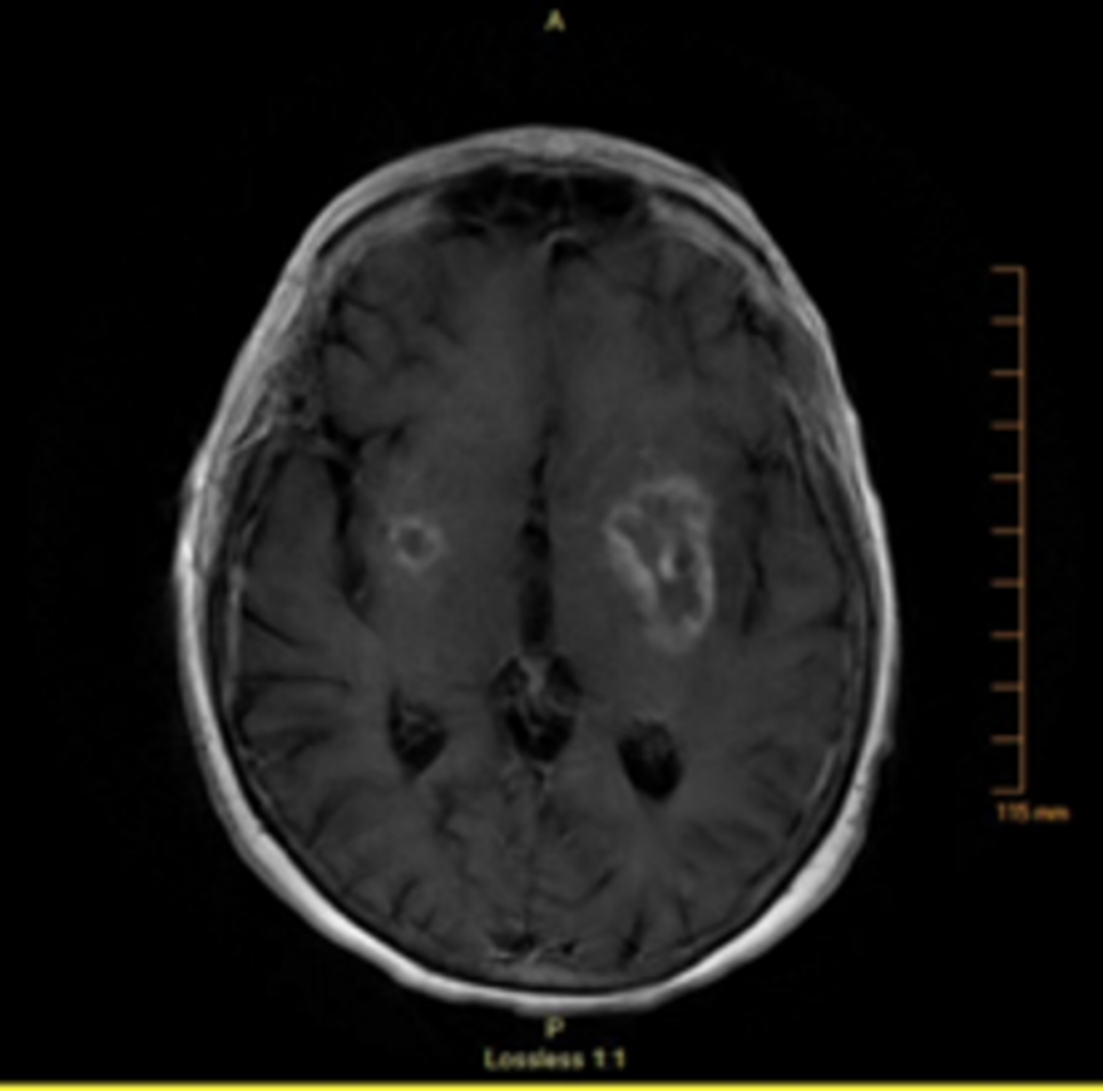
MRI of the brain (T1 fluid-attenuated inversion recovery) - view 1 The imaging revealed a 4 x 2 cm ring-enhancing in the basal ganglia on the left with a small enhancing lesion in the caudate nucleus as well as a small 1 cm round ring-enhancing lesion in the putamen on the right. There were surrounding vasogenic edema and mild mass effect on the left lateral ventricle MRI: magnetic resonance imaging

**Figure 3:**
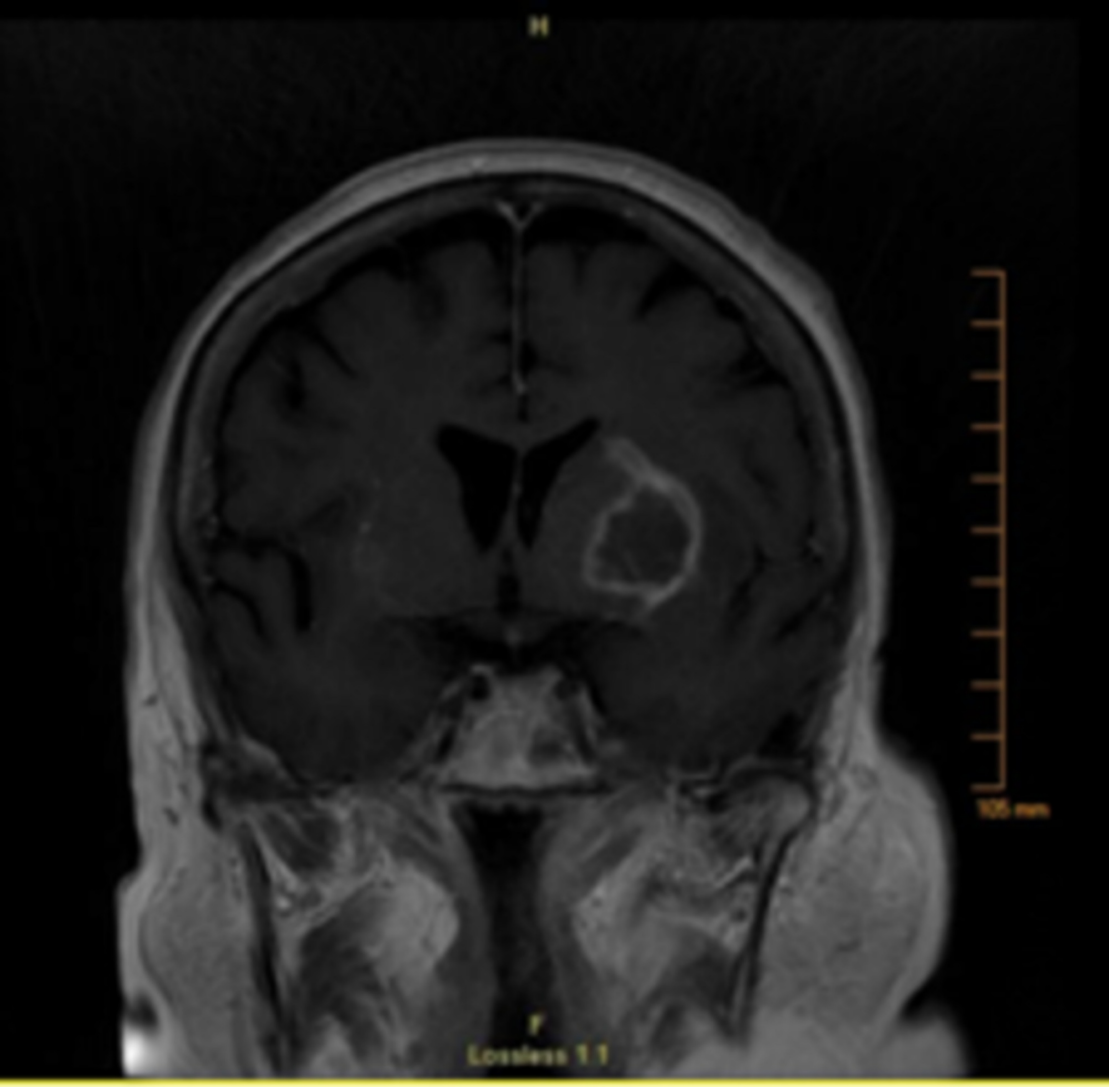
MRI of the brain (T1 fluid-attenuated inversion recovery) - view 2 The imaging revealed a 4 x 2 cm ring-enhancing in the basal ganglia on the left with a small enhancing lesion in the caudate nucleus as well as a small 1 cm round ring-enhancing lesion in the putamen on the right. There were surrounding vasogenic edema and mild mass effect on the left lateral ventricle MRI: magnetic resonance imaging

MRI of the brain with and without contrast revealed bilateral ring-enhancing lesions in the basal ganglia (left larger than right) with surrounding vasogenic edema and mild mass effect on the left lateral ventricle from the larger lesion as shown in Figures 2, 3. CT of the chest, abdomen, and pelvis with and without contrast was performed and was negative for primary malignancy.

Due to the characteristics of the lesions on MRI, there was high suspicion for brain metastases, which prompted a brain biopsy of the left intracranial intraparenchymal mass for diagnosis. The tissue specimen showed necrotic brain tissue with patchy marked inflammatory changes as well as structures morphologically compatible with tachyzoites of toxoplasmosis. Special stains for gram-positive and gram-negative bacteria, fungi, acid-fast bacilli, cytomegalovirus, and herpes simplex virus 1 and 2 were all negative. Immunohistochemistry for toxoplasma was positive and a diagnosis of cerebral toxoplasmosis was made. Serology revealed that the anti-toxoplasma immunoglobulin G was >400 IU/mL (positive). The patient had a negative human immunodeficiency virus (HIV) antigen and antibody screen as well as a normal T cell count (T cell total 1,499 and CD4 786). The patient was started on dexamethasone due to cerebral edema and her immunosuppressive medications were discontinued. As pyrimethamine was unavailable, treatment began with high-dose TMP-SMX (400 mg q12h). Due to an unfortunate post-biopsy hemorrhage, the patient’s neurologic status after treatment initiation was unable to be accurately monitored. The patient was stabilized and transitioned to a long-term acute care hospital with a six-week total course of high-dose TMP-SMX.

## Discussion

Upon review of published literature, we found nine other case reports of RA patients on immunosuppressive therapy who subsequently developed toxoplasmosis; the details are summarized in Table 1.

**Table 1:** Reported cases of non-HIV rheumatoid arthritis patients on immunosuppressive therapy diagnosed with toxoplasmosis RA: rheumatoid arthritis; TNF: tumor necrosis factor; PCR: polymerase chain reaction; CSF: cerebrospinal fluid; TMP-SMX: trimethoprim-sulfamethoxazole; BID: bis in die; HIV: human immunodeficiency virus

Study	Patient no	RA therapy	Toxoplasmosis site	Method of diagnosis	Toxoplasmosis treatment	Prognosis
Matsuura et al. [5]	1	Methotrexate, infliximab, leflunomide, prednisone	Eye	PCR of aqueous humor	Pyrimethamine, folinic acid, sulfadiazine	N/A due to scars from chorioretinitis
Matsuura et al. [5]	2	Methotrexate, etanercept, prednisone	Eye	Fluorescein angiography	Pyrimethamine, folinic acid, sulfadiazine	Improved
Young et al. [6]	3	Methotrexate, infliximab, leflunomide, prednisone	Brain	Brain biopsy	Pyrimethamine, folinic acid, dapsone (sulfa allergy)	Improved
Pulivarthi et al. [7]	4	Methotrexate, infliximab	Brain	Brain biopsy	Pyrimethamine, leucovorin, clindamycin (sulfa allergy)	Unknown
Nardone et al. [8]	5	Adalimumab	Brain	Brain biopsy, PCR of brain tissue	Pyrimethamine, folinic acid, sulfadiazine	Improved
Lewis et al. [9]	6	Methotrexate, prednisolone	Brain	PCR of CSF	TMP-SMX then pyrimethamine and sulfadiazine	No change
Baddley et al. [10]	7	Methotrexate	Brain	PCR of CSF	TMP-SMX (360 mg/day; treatment of choice not approved in Japan)	Improved
Walkden et al. [11]	8	Adalimumab, prednisone	Eye	PCR of aqueous humor	TMP-SMX, clindamycin	Worsened
Bach et al. [12]	9	Methotrexate, anti-TNF prednisolone	Brain	Brain biopsy	TMP-SMX, leucovorin, atovaquone	Improved
Present case	10	Methotrexate, infliximab	Brain	Brain biopsy	TMP-SMX (400 mg BID)	Unknown

Similar to the patient in this case report, these patients were all HIV-negative and had no other risk factors affecting their immune systems. Three of these case reports had eye involvement and six cases had neurologic manifestations [5-12]. Of the nine case reports reviewed, seven were on tumor necrosis factor inhibitors at the time of toxoplasmosis diagnosis [5-8,11,12]. Only one case report described treatment with methotrexate alone [10]. Of the toxoplasmosis encephalitis cases, four were diagnosed by brain biopsy and four by PCR of the CSF. A majority of these cases revealed treatment of toxoplasmosis with pyrimethamine and sulfadiazine plus folinic acid for at least four to six weeks, with mixed results on the prognosis.

This case report also reveals the complications involved in diagnosing toxoplasmosis encephalitis. As previously mentioned, toxoplasmosis encephalitis is definitely diagnosed via PCR of the CSF or brain biopsy [1,3]. PCR had been previously found to lack sensitivity and thus a direct visualization with biopsy specimen was favored; however, new-generation PCR techniques have allowed the diagnosing method to gain popularity [2,4]. The complications of the brain biopsy as seen in this case support the use of PCR testing of CSF as a safer method in lieu of direct visualization on brain biopsy [5]. As our patient suffered from a post-biopsy hemorrhage, which negatively affected the outcome and prognosis of her infection, we are not in a position to make a definitive statement on the effectiveness of treatment and discontinuation of immunosuppressants.

Upon an exhaustive literature search, there were no current recommendations found in regard to treatment length in HIV-negative, non-transplant immunocompromised individuals [6-8]. As the use of biologics in the treatment of inflammatory disorders continues to rise, the risks of opportunistic infections, including toxoplasmosis, needs to be taken into consideration in this subset of patients [9,10]. Due to the large gaps in knowledge about this specific subset of patients, there is a need for further data and recommendations regarding toxoplasmosis diagnosis, treatment, and prophylaxis in this population. In a recent review, 46% of RA patients studied were found to be seropositive for toxoplasma, raising concerns about the potential risk of reactivation with immunosuppressants in this population [13]. Taking into account the lack of data on this subject, there could be potential benefits from serologic testing prior to the initiation of immunosuppressive therapy such as TNF-a inhibitors.

## Conclusions

This case report highlighted the potential role that immunosuppressive therapy plays in HIV-negative, non-transplant immunocompromised patients diagnosed with toxoplasmosis. Upon review of literature, seven similar published cases were found involving RA patients on immunosuppressive therapy, all of whom had no other factors affecting their immune status. As a majority of the case reports cited involved both methotrexate and TNF-a inhibitor therapy, it is unclear as to which medication is responsible for the reactivation of toxoplasmosis in the patient in this case report. However, with only one case indicating that methotrexate alone caused the reactivation of toxoplasma, there is a possibility that the infliximab exclusively was the predisposing factor in the above patient. Continued investigations regarding the necessity of pretesting and/or prophylaxis, particularly in the RA population, will provide insight into treatment modalities in a population at risk for cerebral manifestations of toxoplasmosis.
